# Efficacy of local convection enhanced delivery of chemotherapy using an intracerebral osmotic pump in a rat model of glioblastoma

**DOI:** 10.3389/fonc.2026.1775053

**Published:** 2026-03-04

**Authors:** John McDaid, Julian E. Bailes, Neilank K. Jha, George Bobustuc, Reed Berlet, Cassidy Kessinger, Eliana Whitcomb, John M. Lee, Daniil P. Aksenov, Matthew Walker, Azur Azapagic, Jade Bookwalter, Ata Ullah, Himanshu Sant, Jill Shea, Bruce K. Gale

**Affiliations:** 1 Department of Neurosurgery, Endeavor Health, Evanston, IL, United States; 2 Pritzker School of Medicine, Chicago, IL, United States; 3 Pritzker School of Medicine, University of Chicago, Chicago, IL, United States; 4 Deep Brain BCI Corp, Wilmington, DE, United States; 5 Intent Medical Group, Endeavor Health Advanced Neurosciences Institute, Arlington Heights, IL, United States; 6 Department of Pathology, Endeavor Health, Evanston, IL, United States; 7 Department of Radiology, Endeavor Health, Evanston, IL, United States; 8 Department of Anesthesiology, Endeavor Health, Evanston, IL, United States; 9 Department of Mechanical Engineering, The University of Utah, Salt Lake, UT, United States; 10 Department of Biomedical Engineering, The University of Utah, Salt Lake, UT, United States; 11 Department of Chemical Engineering, The University of Utah, Salt Lake, UT, United States; 12 Department of Surgery, University of Utah School of Medicine, Salt Lake, UT, United States

**Keywords:** brain, carmustine, direct delivery, osmotic pump, resection, temozolomide, tumor

## Abstract

**Background:**

Modern protocols for the treatment of glioblastoma multiforme (GBM) involve resection surgery, followed by chemotherapy and radiation therapy and subsequently adjuvant chemotherapy. While modestly successful in prolonging overall survival, peripherally administered chemotherapy drugs have limited ability to cross the blood brain barrier (BBB), limiting their bioavailability, and thus efficacy, at the tumor site. One way of circumventing the BBB is direct delivery of chemotherapy to the tumor site. Direct application of chemotherapy into the resection cavity during surgery in the form of carmustine/bis-chloroethylnitrosourea (BCNU) wafers has had limited success, in part due to the need for wafer solubilization, which restricts drug distribution and efficacy. The primary limitation, however, is that the drug is only distributed over short distances, for a short time.

**Methods:**

In this study, we evaluated the efficacy of drug perfusion into the tumor resection cavity in a rat glioma model through convection enhanced delivery (CED), using an implanted microfluidic osmotic pump. We compared the effects of two alkylating agents, BCNU and temozolomide (TMZ), on tumor recurrence and survival.

**Results:**

Using pumps containing a high concentration of ferumoxytol — a superparamagnetic iron oxide nanoparticle (SPION) — tissue perfusion was demonstrated *in vivo* by MRI and by post-mortem histology, confirming the effectiveness of the microfluidic pump as a drug delivery device. When delivered by implanted pumps, BCNU (4mg/ml) showed significantly greater efficacy against tumor recurrence than either TMZ; 2-4mg/ml or control (a low concentration of SPION).

**Conclusion:**

BCNU may be an effective choice for CED-driven, locally delivered chemotherapy in GBM.

## 1 Introduction

Glioblastoma multiforme (GBM) is the most common form of primary malignant brain tumor in adults, with a very dismal prognosis; the average survival time following diagnosis is approximately 14 months (1) and the 5-year survival rate is approximately 7% (2). The primary treatment method for GBM is surgical removal, followed by chemoradiation and chemotherapy consolidation (3); however, glioma cells typically infiltrate adjacent tissue, and, despite the best efforts of the surgeon, these residual cells rapidly initiate tumor regrowth. Although there is no standard of care for recurrent GBM, subsequent treatment may involve additional surgery, chemotherapy, and/or radiation (4). GBM is difficult to treat with conventional chemotherapy, administered orally or intravenously, as most drugs are unable to cross the blood-brain barrier (BBB) in adequate concentrations to achieve therapeutic levels at the tumor site (5, 6). Chemotherapeutic drugs generally exhibit a short serum half-life and require high doses to achieve therapeutic levels, which increases the risk of systemic toxicities and adverse effects (7).

Many methods to bypass the BBB have been explored, with mixed results (8–10). One approach is to deliver the drug directly to the tumor, or to the peritumoral space, during tumor resection, thus taking advantage of direct surgical access. Local application of the alkylating agent BCNU (1,3-bis(2-chloroethyl)-1-nitrosurea) in the form of Gliadel^®^ Wafers, which are implanted into the tumor cavity, is already FDA approved (11). The drug is embedded within a hydrophobic copolymer matrix that facilitates drug release as it degrades (12). While the use of these biodegradable polymers has a low toxicity profile, their efficacy is limited, as most drug is released within a week of implantation (13, 14), and diffusion distance is only a few millimeters. In addition, such devices are associated with edema and infection, and with diminished efficacy over time, possibly due to limited, passive drug diffusion and depletion by CSF drainage. The use of convection-enhanced delivery (CED) may allow for more efficient, directional drug delivery over greater distances, and for longer time periods, and thus target the residual tumor tissue, beyond the margins of the resection cavity (15–23). In this study, we examined the effects of targeted chemotherapeutic drug delivery, utilizing the principles of CED via an implanted microfluidic pump, on tumor regrowth after surgical resection in a rat model of GBM.

## 2 Methods

### 2.1 Animals

Adult male Sprague Dawley rats (250-300g) were obtained from Charles River Laboratories. All animal procedures were conducted following protocols approved by the Institutional Animal Care and Use Committee (IACUC) at the Endeavor Health Research Institute, ensuring compliance with applicable ethical guidelines. Adjustments were made to ensure consistency in animal size/weight at the time of procedures. A total of 17 rats were used in this study.

### 2.2 Microfluidic osmotic pump design

**Figure 1:**
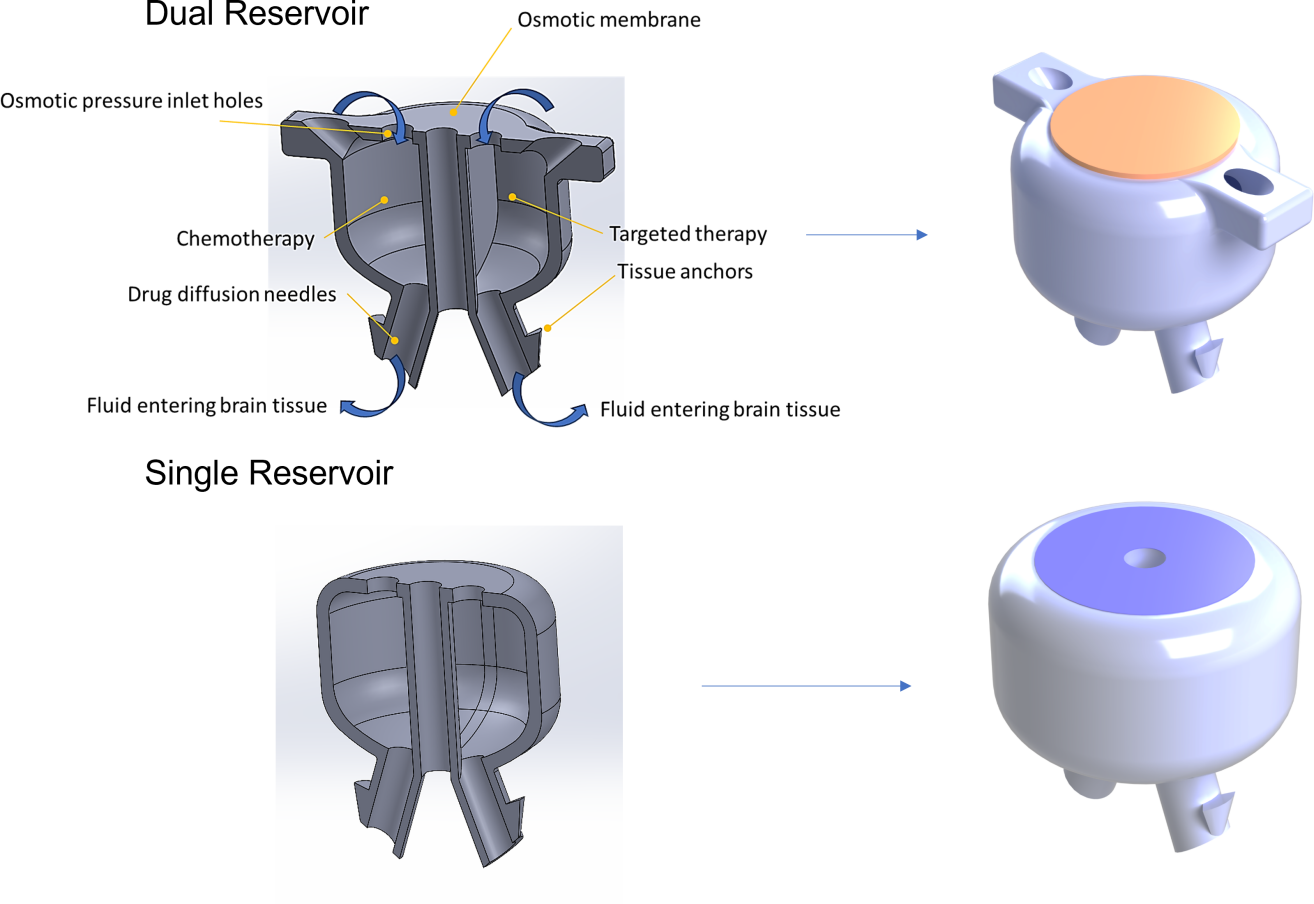
Illustration of the engineering design and brain-targeted mechanism of the intracranial drug delivery pump. Three-dimensional schematics of an implantable drug pump design showing internal structure and flow pathways for targeted drug diffusion. These implantable pumps include either a single or dual reservoir that is enclosed by a biocompatible housing. Pump reservoirs are filled immediately before implantation, either via the vent holes for the dual reservoir or one of the perfusion needles for the single reservoir pump. Once the pump is filled, a semipermeable osmotic membrane is placed on top of the pump, over the inlet holes. When the pump is implanted, osmotic pressure initiates interstitial water flow through the semipermeable membrane, generating hydrostatic pressure that drives the drug solution in the internal reservoir(s) out through the needles at the base of the device into surrounding tissue (indicated by blue arrows). The needles are strategically positioned to ensure even distribution of drug release into the surrounding tissue. The pump is secured in place in the resection cavity by tissue anchors. The osmotic pressure–driven flow ensures stable, uniform drug release through the needles, optimizing delivery efficiency.

We have previously described the design and testing of an implantable microfluidic osmotic pump (24), which, when placed in the resection cavity, enables perfusion of local brain tissue through CED (25). The 3D-printed device (24, 25) (**Figure 1**) was designed to achieve controlled drug dosage and targeted delivery, while minimizing drug loss to other areas.

Pumps were 3D-printed from acrylonitrile butadiene styrene (ABS)-like thermoset MicroFine™ resin on a high-resolution stereolithography printer (Protolabs Inc.). The devices had either one or two independent reservoirs (pump dimensions X: 3.60mm Y: 4.01mm Z: 3.60mm, or X: 5.09mm Y: 4.01mm Z: 3.60mm, respectively). The dual-reservoir version allows for simultaneous delivery of two distinct therapeutic agents—cytotoxic or immunologic, individually or in combination, to enable customized drug combinations and dosages. For both types of pumps, pores at the top of each drug reservoir were covered by a one-way osmotic membrane (Millipore™ membrane filters, pore size 25nm), made from hydrophilic, biologically inert mixed cellulose esters (cellulose acetate and cellulose nitrate), which are permeable to water from the interstitial fluid. The bottom of each reservoir had two smooth, small-bore micro-perfusion needles, designed to be placed within the resection cavity walls, directly into the brain parenchyma up to a depth of 3-5mm, with minimal trauma to healthy brain tissue.

**Figure 2:**
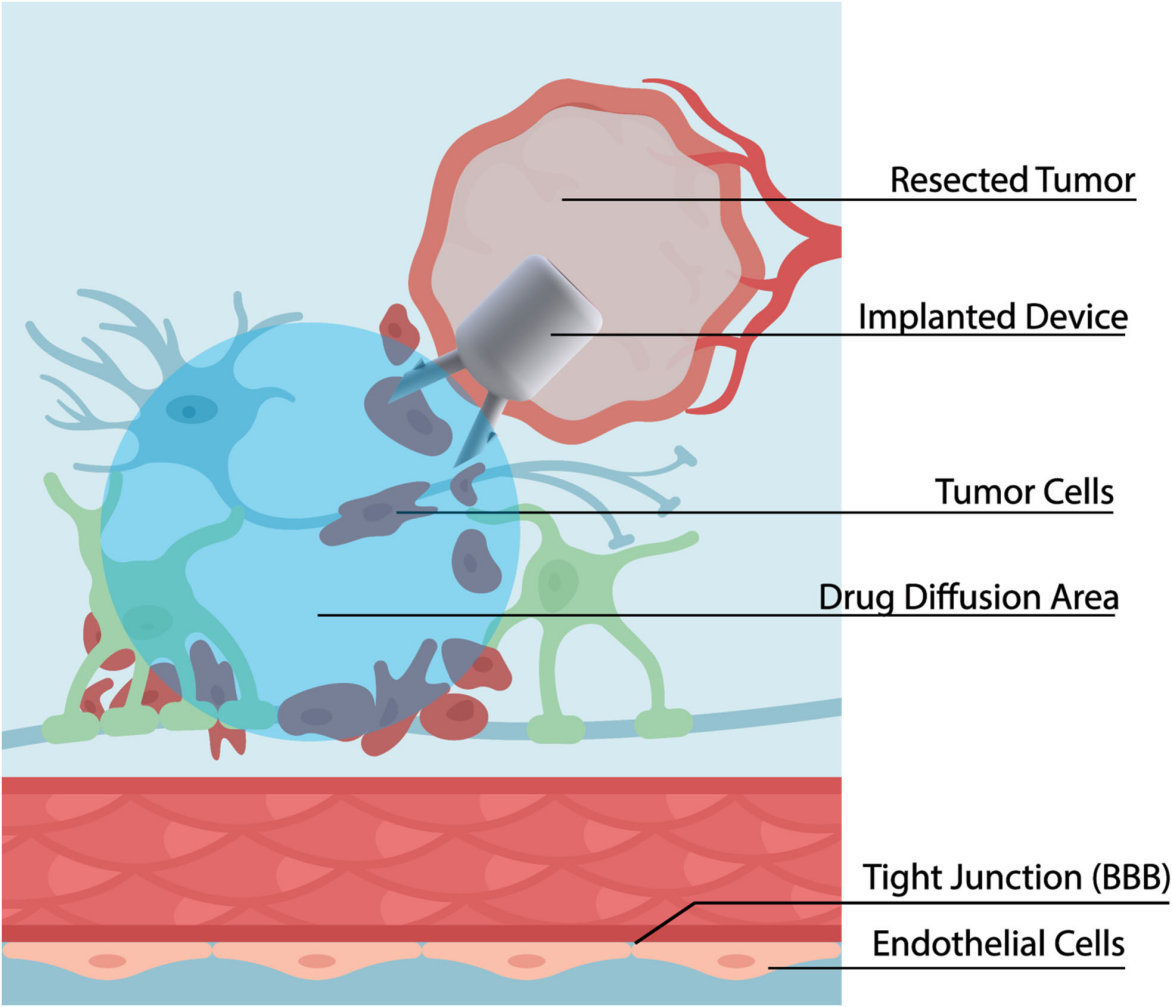
This diagram illustrates a strategy to combat brain tumor recurrence by delivering therapeutic agents directly to the tumor microenvironment following surgical resection. The resected tumor cavity, created after bulk tumor removal is outlined in red. Highly invasive tumor cells (dark gray/purple) often remain embedded within the surrounding healthy brain parenchyma (light blue). To address these residual cells and overcome the blood-brain barrier (BBB) — formed by endothelial cells with tight junctions — a pump (Implanted Device) is placed in the resection cavity after surgical removal of the tumor. The pump continuously releases a therapeutic agent, creating a local drug diffusion area (blue shaded region) where high drug concentrations are achieved, targeting the residual, invasive tumor cells while minimizing systemic exposure.


When the drug-filled pump is implanted in the resection cavity (**Figure 2**), the hyperosmolar drug solution inside the reservoir creates osmotic pressure that continuously draws water from brain interstitial fluid into the reservoir, through the osmotic membrane. According to Van’t Hoff’s law:

Π=i·C·R·T

the osmotic pressure ( Π) is proportional to solute concentration (*C*), temperature (*T*), and the gas constant (*R*); *i* reflects the degree of solute ionization. As water is drawn into the pump through osmosis, it generates a hydrostatic pressure inside the reservoir that drives the drug solution out through the perfusion needles. Drug perfusion into brain tissue occurs through hydrostatic pressure-driven bulk flow (CED) during active drug delivery, with diffusion (driven by concentration gradient) occurring concurrently and becoming more dominant at the periphery and after drug delivery is complete.

### 2.3 C6 glioma cell culture

Rat C6 Glioma cells (American Type Culture Collection (ATCC), Cat. No. CCL-107) were cultured in FK-12 medium supplemented with penicillin/streptomycin (1%) and fetal bovine serum (2.5%), in 35mm x 10 mm Corning cell culture dishes, and maintained at 37 °C in a humidified atmosphere with 5% CO_2_ and 95% air. Cells were subcultured every 2–3 days at a 1:2 to 1:3 ratio. The cell monolayer was washed with 0.25% Trypsin-EDTA solution to detach cells, which were subsequently reseeded in fresh medium. C6 glioma cells were prepared for implantation once they reached 60-70% confluence in the log growth phase. Prior to implantation, cells were washed with 350µL of phosphate-buffered saline (PBS) and incubated with 200µL of trypsin-EDTA. After 5 minutes, cells were centrifuged at 125g for 5 minutes and the cell pellet resuspended in Hank’s buffered salt solution. The suspension was then transferred into a sterile 1mL syringe attached to a vinyl catheter tube, ensuring air bubbles were minimized.

### 2.4 C6 glioma cell injection

Brain tumors were induced by sub-cortical injection of C6 glioma cells, which results in reliable tumor formation that can be visualized using MRI (26). Animals were anesthetized using 5% isoflurane and placed in a stereotactic frame under continuous anesthesia (1.5-3.0% isoflurane). The head was shaved, and the scalp sterilized with povidone-iodine and an alcohol wipe. Preoperative analgesia consisted of ketoprofen (5mg/kg, s.c.) and lidocaine (4mg/kg, s.c. given under the scalp). A midline incision was made to expose the skull and a 1mm diameter drill hole was made in the skull using a dental drill, according to the following stereotactic co-ordinates, relative to bregma, anterior-posterior +2mm, medial-lateral +2mm, dorsal-ventral -2.5mm. An injection cannula (0.31mm outer diameter, P1 Technologies), attached by a vinyl tube to a Hamilton glass syringe with a 25-gauge Hamilton needle, was lowered into the cortex, through the drill hole, while attached to a cannula holder on the stereotactic frame. 10^4^ C6 glioma cells suspended in 10 μL medium were injected at a 2.5mm depth from dura at a rate of 2μL/min, using a syringe pump. After infusion was complete, the drill hole was sealed using a wax plug and the skin sutured back over the skull using interrupted monofilament sutures. Rats were monitored postoperatively and provided with analgesia (ketoprofen, 5 mg/kg s.c. 24h later) and supportive care.

### 2.5 MR imaging

MR imaging was performed approximately 7 days after C6 glioma cell infusion and then approximately every 3 days to monitor tumor growth until tumors were deemed large enough to allow resection and pump implantation within the resection cavity margins. MRI was performed on a 9.4 T imaging spectrometer (BioSpec 94/30USR, Bruker BioSpin MRI GmbH). Axial T2-weighted images were acquired using a multi-spin-echo (RARE/fast spin-echo) sequence (TR 2000–5000 ms; effective TE 26–60 ms) with a field of view of 20 mm x 20 mm, an in-plane matrix of 128–256 x 128-192, and a slice thickness of 0.75 mm. Axial T1-weighted images were acquired using a gradient-echo FISP sequence with inversion preparation (TI 300 ms; TR 3.7-7.7 ms; TE 1.5 ms; flip angle 10 degrees) using the same geometry. To assess tissue perfusion from the pump, some pumps were filled with ferumoxytol (ultrasmall superparamagnetic iron oxide nanoparticles; SPIONs), and the spatial extent of SPION-related signal change was measured using the region-of-interest (ROI) tool in ParaVision (Bruker). Experimenters were blinded to treatment groups for review of MRI data.

### 2.6 Tumor resection

Resection was performed as described in Berlet et al. and Bastiancich et al. (25, 27). Briefly, a 5mm craniectomy was performed around the original injection site, exposing the dura. Tumor mass was removed using a combination of suction and irrigation. Hemostasis was achieved with bipolar cautery (Surgicell Fibrillar), topical hemostatic agents, and saline irrigation. After placement of the pump, the resection cavity was covered with a dural graft matrix (DuraGen, Integra LifeSciences), and the scalp was closed with sutures. Rats were monitored postoperatively and provided with analgesia (ketoprofen, 5 mg/kg s.c. 24h later) and supportive care.

### 2.7 Pump preparation and implantation

**Figure 3:**
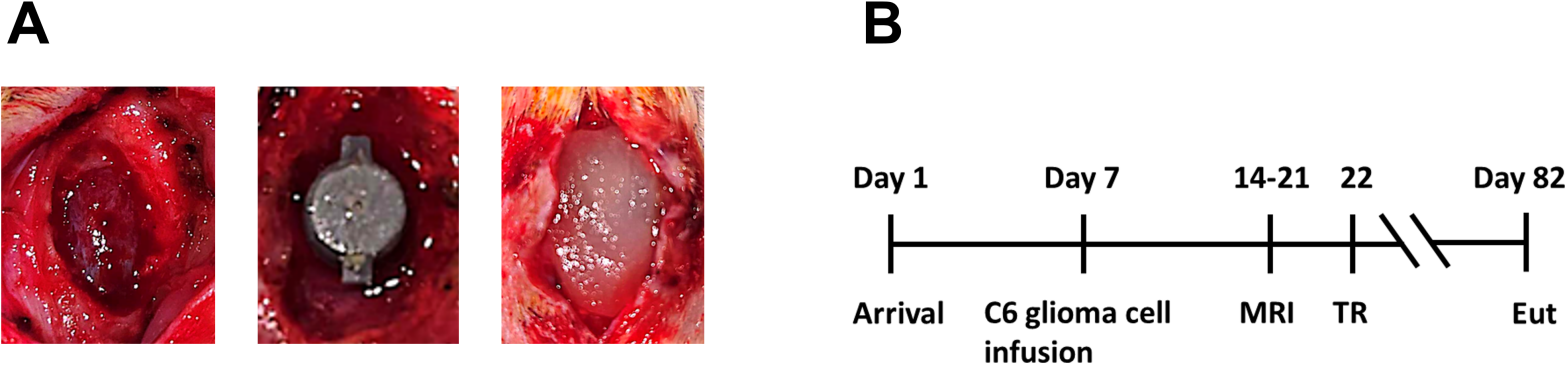
Surgical implantation of drug delivery pump and experimental workflow in a rat glioma model. **(A)** Representative intraoperative images illustrating the sequential steps of intracranial drug pump implantation in a rat glioblastoma model. (Left) Complete resection of the tumor exposes a cranial cavity, creating a space for pump placement. (Middle) The pump is positioned within the cavity to enable localized therapeutic infusion. (Right) The surgical site is covered by a dural graft matrix (DuraGen) to secure the device and establish a stable barrier between the implanted pump and overlying skin. **(B)** Experimental timeline. Animals arrived on Day 1 and received an intracranial infusion of C6 glioma cells on Day 7 to induce tumor formation. Magnetic resonance imaging (MRI) was conducted intermittently to confirm tumor development. Once tumors reached an appropriate size, tumor resection (TR) was performed followed by pump implantation for targeted drug delivery. Animals were monitored postoperatively for recovery and tumor recurrence, and healthy animals underwent euthanasia (Euth), typically 60 days after TR, for endpoint analysis.

Prior to implantation, pumps were sterilized using the advanced cycle on the STERRAD 100NX hydrogen peroxide gas plasma sterilization system, at temperatures between 45 °C to 55 °C, for 45 to 55 minutes, with a sterilant concentration of approximately 59% hydrogen peroxide. STERRAD-compatible packaging materials were used to ensure optimal sterilization efficacy and material integrity. Pump reservoirs were filled immediately before implantation under sterile conditions using silicone tubing attached to the vent holes, allowing air to escape through the perfusion needles. The vent holes were then sealed using a small amount of bone wax. Dual reservoir pumps were either preloaded with SPIONs (0.2 µg/ml in 10 X Tris-buffered saline), or the alkylating agent BCNU (4mg/ml in H2O). Single-reservoir pumps were filled with temozolomide (2 or 4 mg/ml in H2O), or BCNU (4mg/ml in H2O). While both drugs have relatively low water solubility, we chose not to use non-polar solvents, to avoid precipitation of drug in the pump or needles when drug solution interacted with interstitial water. Two rats received dual-reservoir pumps preloaded with a higher concentration (20 µg/mL) of SPIONs in 10X Tris-buffered saline to enable monitoring of perfusion from the pump with MRI, as previously reported (25). Each pump contained only one drug; dual reservoir pumps had the same drug in both reservoirs. During resection surgery, a pump containing a drug or SPIONs was inserted into the resection cavity without pressure, so as to minimize any damage to the brain parenchyma (**Figure 3A**). Rats (n=17) received pumps containing either low concentration SPIONs (n=4), high concentration SPIONs (n=2), BCNU (n=6) or TMZ (n=5). All pumps were implanted with the perfusion needles aligned along the sagittal plane of the resection cavity for optimal MRI imaging. Rats were euthanized when tumor regrowth impacted the animal’s well-being; surviving rats were euthanized 2–3 months after pump implantation (**Figure 3B**).

### 2.8 Histology

Animals with an implanted pump containing 4mg/ml of BCNU (n=6) or 2 or 4 mg/ml of TMZ (n=5, pooled) were sacrificed 18–92 days or 17–56 days after tumor resection, respectively, depending on their state of health. Euthanasia was performed by decapitation with a sharpened guillotine, per Endeavor Health IACUC Policy, under deep anesthesia using isoflurane inhalation (5% with 0.5-1.0 L/min mixed air). Brains were extracted and, after pumps were removed, were post-fixed overnight in 4% PFA. Coronal brain slices (5–10 µm) encompassing the resected tumor site/pump placement area and tumor slices were prepared using a standard paraffin-embedding procedure. Slices were stained with hematoxylin and eosin (H&E). Tumor tissue was examined histologically to confirm C6 glioma characteristics; that is, to assess overall tumor histological architecture, parenchymal infiltration, and/or necrosis; and to determine tissue effects of iron oxide, BCNU or TMZ. The extent of inflammatory cells, surrounding gliosis, and any other pathological findings were recorded. Prussian blue staining was used to identify ferric iron from pumps filled with high concentration SPIONs for comparison with MRI images. All stained sections were independently reviewed by a board-certified neuropathologist who was blinded to the treatment assignments.

## 3 Results

### 3.1 Tumor recurrence and survival

The primary goal of this study was to evaluate tumor recurrence and survival in a rat model of GBM, after local delivery of the chemotherapy drugs BCNU or TMZ. In our experience using this model, tumor growth, determined using MRI, is rapid, relentless, and invariably results in death within days or weeks. After tumor growth, we followed the standard clinical approach of resection surgery, followed by placement of an osmotic microfluidic pump in the resection cavity for local chemotherapy delivery. The feasibility of this approach was determined in a previous study (25) where MRI imaging showed that the SPION was distributed several millimeters from the pump. Because clinically, over 80% of human GBMs recur within 2cm of the initial tumor, we expected that drugs delivered using this approach would reach residual tumor tissue, thus preventing tumor recurrence (28, 29).

**Figure 4:**
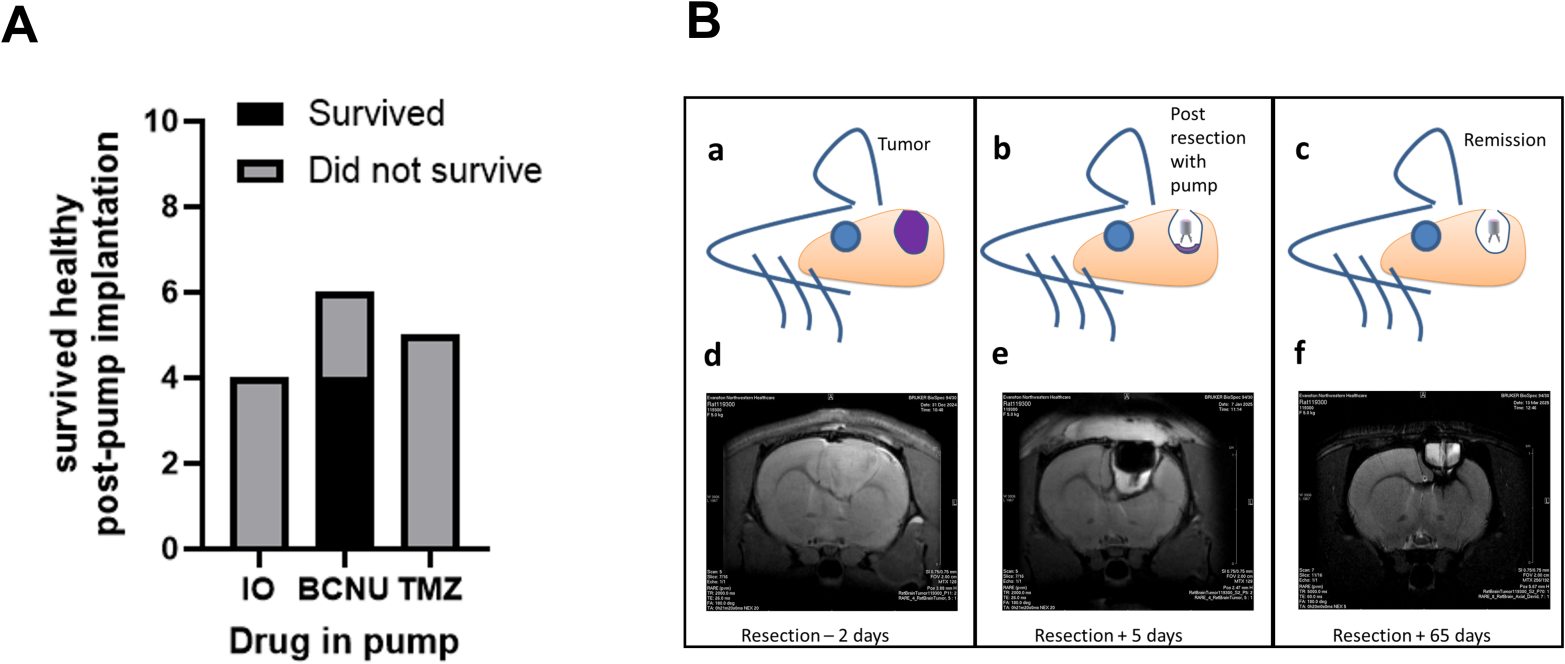
Survival outcomes following pump implantation with different drug treatments in a rat glioma model. **(A)** Bar graph illustrates the number of rats that survived or did not survive following intracranial pump implantation with iron oxide (IO), BCNU, or temozolomide (TMZ). Statistical analysis using a Chi-squared analysis demonstrated a significant improvement in survival in the BCNU-treated group compared to IO or TMZ. This suggests that BCNU is better tolerated and more effective in maintaining postoperative survival following intracranial drug delivery. These findings indicate that BCNU may provide a safer and more efficacious therapeutic option for localized chemotherapy via implanted pumps. **(B)** Illustration of tumor growth and surgical resection with an intracranial drug delivery pump in a rat model using serial MR imaging. Schematic diagrams of experimental stages: (a) pre-treatment intracranial tumor (purple), (b) post-surgical resection and placement of an intracranial drug delivery pump with drug (BCNU) and (c) Long-term outcome following intervention which, in this animal, led to remission. (Bottom) Corresponding axial T2-weighted images: (d) Pre-resection scan (2 days before resection) showing a T2-hyperintense intraparenchymal mass with surrounding edema and mass effect. (e) Early postoperative scan (5 days after implantation of pump filled with BCNU) demonstrating the resection cavity and catheter/pump trajectory; fluid within the cavity/reservoir appears T2-hyperintense, with residual postoperative edema. (f) Late follow-up scan showing decreased cavity size, normalization of mass effect, and no discrete T2-hyperintense mass at the sensitivity of this sequence. Acquisition: slice thickness 0.75 mm, FOV 20 mm, matrix 128. Scale shown on the right margin (mm).

Of the 4 rats that received pumps filled with a low concentration of SPIONs, none survived, with most euthanized due to the effects of tumor regrowth. Of the 6 rats that received BCNU, 4 survived to the study endpoint, apparently healthy and with no MRI-detectable tumor, while 2 were euthanized due to tumor regrowth. Of the 5 rats that received TMZ (2 or 4 mg/ml), none survived, with all 5 euthanized due to tumor regrowth (**Figure 4A**). MRI scans from animals that survived showed evacuation of reservoir contents and tissue recovery, including a reversal of the midline shift caused by the tumor (**Figure 4B**).

Use of a Chi-squared analysis revealed a significant effect of BCNU, compared to SPIONs (χ^2^ (1, 10) = 4.44, p = 0.03) and TMZ (χ^2^ (1, 11) = 5.23, p = 0.02), indicating that use of BCNU in the osmotic pump was more likely to have a favorable survival outcome than SPIONs or TMZ.

### 3.2 MRI and histology

**Figure 5:**
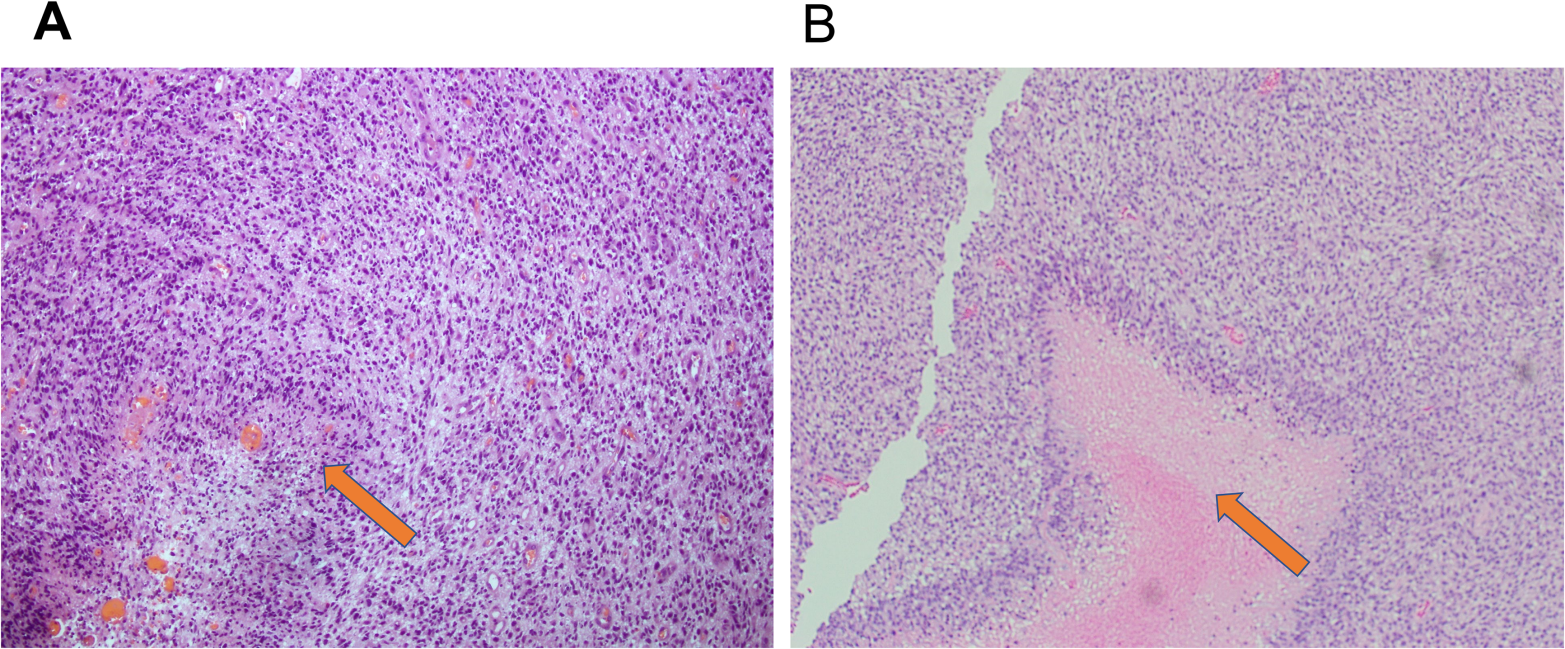
Representative hematoxylin and eosin (H&E) stained sections showing comparative histopathology of human glioblastoma and C6 rat glioma model. H&E staining provides a general overview of tissue structure allowing distinction between different cell types and tissue features. Hematoxylin stains nuclei blue/purple, showing cell structure and organization. Eosin stains the rest of the tissue pink, showing background architecture. **(A)** Human brain tissue with GBM exhibits hypercellularity as well as an area of focal palisading necrosis (orange arrow). Reactive changes due to an increase in cells after injury is typical of a high-grade malignant tumor. **(B)** Rat brain tissue from a rat C6 glioma model demonstrates a highly infiltrative and proliferative tumor. The orange arrow indicates a region of extensive central necrosis as a component of palisading necrosis, a hallmark often seen in aggressive gliomas, where tumor cells line up in an organized fence-like palisade arrangement around a central area of necrosis. The pale, eosinophilic (pink) area with dead cells and debris reflects necrosis. A dense row of viable tumor cells is seen along the edge of the necrosis area. Overall, the histological analysis confirms that the rat glioma model recapitulates the key morphological features—specifically hypercellularity and the presence of necrosis—characteristic of aggressive human glioblastoma.

Hematoxylin and eosin (H&E) staining and MR imaging demonstrated that the rat glioma model closely replicated the histopathological characteristics of human GBM (**Figure 5**). In human (**Figure 5A**) and rat (**Figure 5B**) brain sections, tumor regions exhibited hypercellularity with densely packed atypical cells with varying shapes and sizes, which is typical of high-grade gliomas. Prominent areas of palisading necrosis (indicated by arrows), where tumor cells align around a central necrotic core, a defining feature of GBM, were evident in the both brain sections. Additionally, areas of microvascular proliferation, hemorrhage, and perivascular crowding were observed in both tissues, further confirming the similarity in tissue microarchitecture. These similarities support the use of the rat glioma model as a valid preclinical platform for investigating glioblastoma pathophysiology and for evaluating the efficacy of targeted chemotherapeutic delivery.

**Figure 6:**
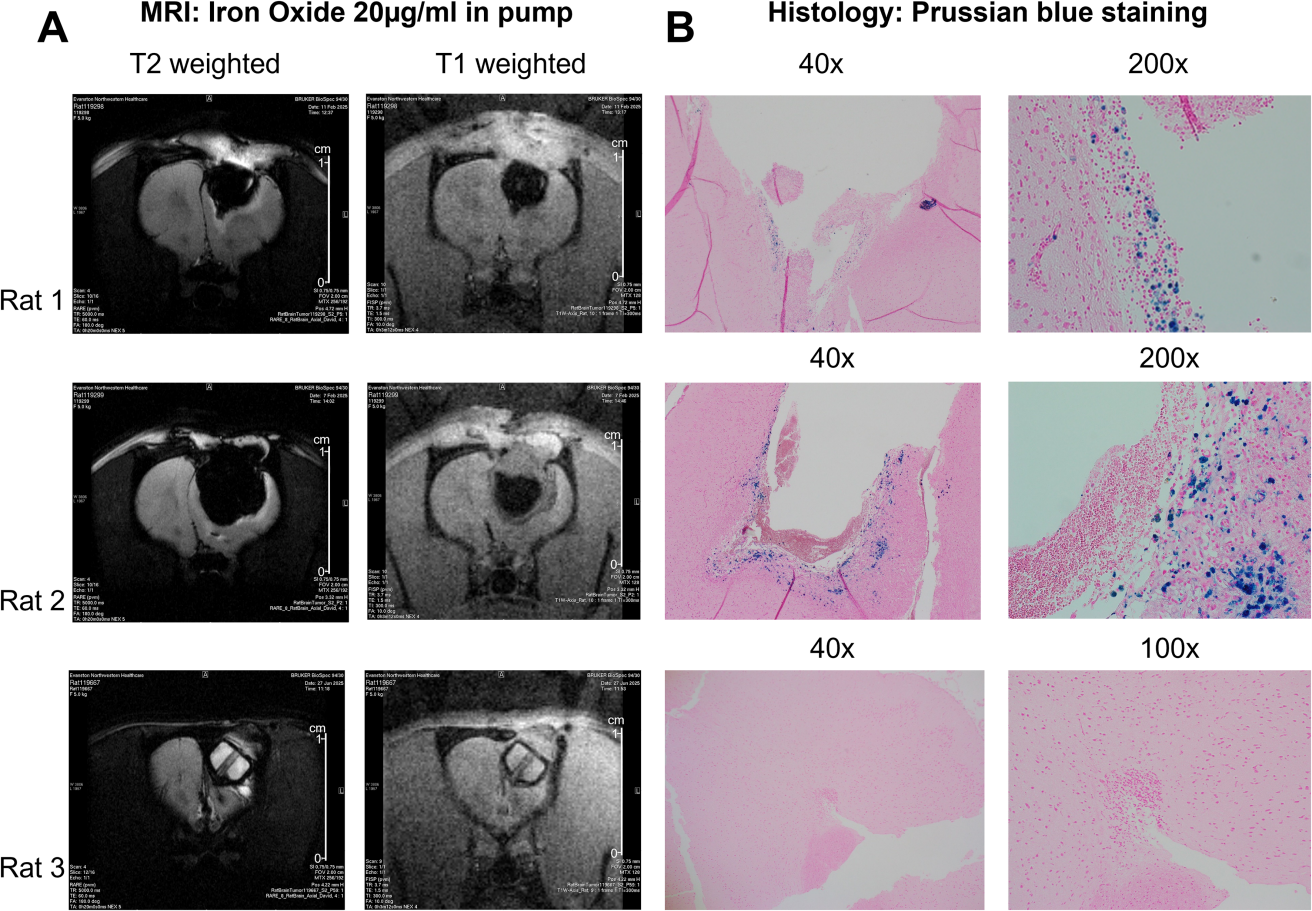
MRI-histology correlation for ferumoxytol (SPION) distribution in a rat C6 glioma model. This figure shows the correlation between MRI and post-mortem histology in 3 rats following insertion of pumps containing 20 µg/mL SPIONs into the GBM resection cavity in the right frontal cortex. For each animal: **(A)** left panels in each row display axial T2-weighted MRI scans and the right panels display axial T1-weighted MRI scans. On T2-weighted images, SPIONs produce marked hypointense (dark) signal due to susceptibility/T2 effects. On T1-weighted images (right panels), the infusion site shows concentration-dependent signal changes (mixed hypo-/hyperintensity), which can help delineate the pump reservoir and adjacent tissue. **(B)** The panels present corresponding Prussian blue-stained histological sections at low (40x) and high (200x) magnification. Prussian blue labels ferric iron (Fe3+) as blue deposits, confirming the presence and localization of SPIONs within the tissue. The magnification used for histological sections is shown above images.

MRI and post-mortem histology were compared to assess tracer release and tissue distribution after pump implantation, using high concentration SPIONs as a surrogate marker. T2-weighted MRI performed after pump implantation revealed marked hypointense (dark) signal adjacent to the resection cavity and extending into surrounding tissue (**Figures 6A**, left panels), consistent with susceptibility/T2 effects from iron oxide. Corresponding T1-weighted images showed mixed signal intensity at the infusion site (**Figures 6A**, right panels), consistent with concentration-dependent relaxation effects of iron oxide and surrounding tissue changes. Histological analysis using Prussian blue staining corroborated the MRI findings (**Figure 6B**), confirming iron deposition within and around the resection cavity and extending into adjacent tissue.

**Figure 7:**
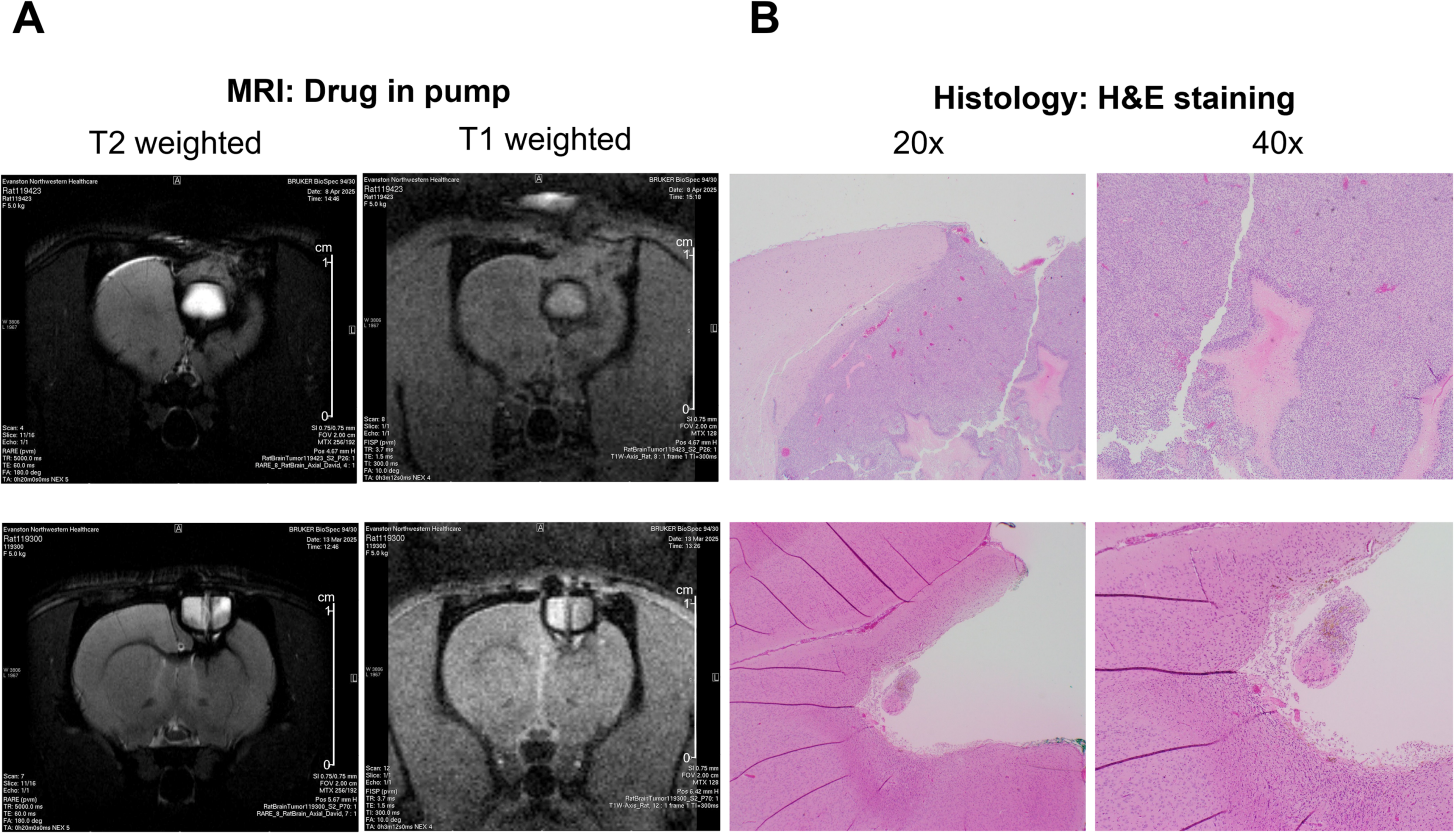
Representative MRI and histological findings after pump-mediated delivery of temozolomide (TMZ) or BCNU in a rat glioma model. Each row shows **(A)** axial T2-weighted (left) and T1-weighted (right) MRI scans and **(B)** corresponding H&E-stained histological sections from a representative animal in each treatment group. The top row shows an animal treated with TMZ in the pump (2 mg/mL). T2-weighted imaging demonstrates a recurrent hyperintense mass/edema with mass effect adjacent to the resection cavity; the fluid-filled pump reservoir is visible within the cavity. H&E staining confirms recurrent tumor with palisading necrosis. The bottom row shows an animal treated with BCNU in the pump (4 mg/mL). T2-weighted and T1-weighted MRI show the postoperative cavity with the pump reservoir in place and no discrete recurrent mass at the sensitivity of these sequences. H&E staining shows a cavity at the infusion site without evidence of residual tumor. The magnification for all histological sections is shown above images.

MRI and H&E staining were used to evaluate postoperative changes and tumor recurrence following pump-mediated delivery of TMZ or BCNU in the rat glioma model. Because TMZ and BCNU are not intrinsically MRI-visible, MRI was used to monitor the resection cavity and pump position, edema/mass effect, and the presence or absence of a recurrent mass. In the representative TMZ-treated animal (**Figures 7A, B**, top row), T2-weighted imaging demonstrated a recurrent hyperintense mass/edema with mass effect adjacent to the resection cavity, and H&E staining confirmed recurrent tumor with necrosis. No difference was seen between animals treated with 2 or 4 mg/ml TMZ. In contrast, a representative BCNU-treated animal (**Figures 7, B**, bottom row) demonstrated resolution of mass effect and no discrete recurrent mass on T2-weighted imaging at the sensitivity of this sequence, with H&E showing a cavity at the infusion site without evidence of residual tumor.

Collectively, these experiments demonstrated that (1) the rat glioma model reproduces human glioblastoma histopathology (2); the pump delivery system enables accurate and localized infusion of agents, directly targeting residual tumor tissue; and (3) MRI serves as a robust, non-invasive tool to visualize and validate intra-tumoral delivery and therapeutic response, which is corroborated by histopathology. This integrated approach provides a strong preclinical platform for testing chemotherapeutic and nanotherapeutic strategies in GBM.

## 4 Discussion

After resection surgery, GBM treatment is usually followed by a course of radiation therapy and oral or intravenous chemotherapy, but these drugs show low efficacy in the prevention of tumor recurrence, mainly due to their low brain bioavailability and high systemic toxicity. While the use of local chemotherapy has been suggested for decades, its effective use has been stymied by complications associated with either open-system fluid dynamics or solid closed systems. Two commonly used chemotherapeutic drugs, TMZ and BCNU, have been administered directly into the tumor resection cavity from implanted devices such as an Ommaya reservoir. While the Ommaya reservoir utilizes positive pressure, drugs are distributed through the ventricular space, thus minimizing drug diffusion and local tissue perfusion. As an open system, this approach also increases the risk of infection (30). BCNU has been used in a closed system as Gliadel wafers, which have demonstrated diffusion of up to 1 cm from the tumor site (31), with tissue concentrations within the millimolar range in animal models of GBM (31), but as most tumors recur within 2 cm of the original resection cavity (32), a significantly increased tissue perfusion distance may be therapeutically necessary. In this study, using a surgically implanted microfluidic pump, we have combined the advantages of local, directional, convection-enhanced drug delivery with those of a closed system, thus maximizing targeted tissue perfusion and minimizing the risk of complications such as infection.

In addition to determining the efficacy of both BCNU and TMZ, we also examined the tissue perfusion of the SPIONs in the immediate vicinity of the resection cavity. The combination of MRI perfusion tracking and histology indicates release of SPIONs from the pump at 2 days, and significant perfusion was still observed at 35 days after resection and pump placement. SPION perfusion was disproportionately detected at the bottom of the resection cavity, indicating directional release of the pump contents. As there may be more residual tumor at resection margins in the lower reaches of the resection cavity, this directionality is encouraging, as it may maximize drug concentration where tumor recurrence is more likely to occur.

Although BCNU and TMZ drugs have both been shown to prolong patient survival in the clinical setting, in this study only BCNU showed efficacy in preventing tumor recurrence and facilitating survival when compared to control. Although differences in efficacy may reflect different bioavailability, stability, or potency (BCNU is a bifunctional alkylator and TMZ is a monofunctional alkylator), both drugs were delivered locally and showed similar solubility in water (up to 4 mg/ml). Both drugs are unstable *in vitro*, with reported half-life values as low as 40 min (33) and 1.8 hours (34), respectively. While active BCNU metabolites are measured in humans for 3–4 days, the active TMZ metabolite MTIC has a half-life of few minutes. TMZ has a reported lower potency of inhibition of GBM growth *in vitro*, compared to lomustine or nimustine, similar nitrosoureas to BCNU (35), but is more effective than BCNU when applied locally, in the form of a polymer wafer, in a rat model of GBM similar to ours (36). The efficacy of BCNU and TMZ may also be differentially affected by the tumor methylation status, specifically with evidence suggesting that MGMT methylation has less of an effect on the efficacy of BCNU (37) compared to TMZ (38). The C6 glioma cell line used in our study has been reported to express high levels of MGMT (39, 40).

### 4.1 Study limitations

This study addressed one of the stated limitations of a previous study (24), namely demonstrating the efficacy of drug distribution from an implanted pump; however, a small number of animals were used, thus further studies are required to confirm and expand on these results. We also did not assess therapeutic effects beyond 3 months after resection surgery and pump implantation, and additional studies with a longer endpoint of at least 6 months are required to evaluate longer-term survival and tumor recurrence over time. We also did not study drug stability in the pump, nor did we confirm the comparative presence of active metabolites or test for the methylation status of MGMT, given the reported high MGMT expression in the C6 glioma cell line implied by others (39, 40), all of which may have accounted for the differences in drug efficacies observed in the study. This study was conducted using only male rats, given that males have a higher prevalence of GBM and poorer outcomes (41, 42). However, future studies should evaluate responses in both male and female models.

While it has been previously shown that drugs can be effectively administered by CED, the engineering science, technology and clinical response have not been fully elucidated. Our study has shown that an indwelling microfluidic pump can effectively deliver drug into the tumor recurrence zone, and that tissue perfusion can be tracked by MRI using iron-oxide labelling. While iron oxide signal intensity and histology can be used to measure pump efficacy and tissue perfusion respectively, this does not allow for measurement of tissue perfusion of actual drug. One possibility is conjugation of drug to iron oxide, but this is not always feasible, with the added complication that tissue perfusion of a drug-iron oxide conjugate may be influenced by the iron oxide moiety.

Use of microdialysis in combination with a convection device/detection of electrical activity (EEG) combination may not be feasible in rats, due to space constraints, but preclinical validation of the current device in larger animal models, such as pigs or monkeys will allow for addition of chronically implanted micro-dialysis probes in the adjacent brain parenchyma, which in turn will allow for simultaneous EEG monitoring and drug/dialysate sampling from the resection cavity and its environs. An alternative, space saving technique such as fast-scan cyclic voltammetry may also be employed in the future, at least for measurement of BCNU, which has a distinct redox profile, using chronically implanted carbon fibers (43).

The stability of chemotherapeutic drugs in aqueous solution over time is a known to be low. While we did not test the stability of the drugs used in the pump, we recognize this to be a potential clinical limitation. Future studies will test the use of solid drug, inserted into the pump as a pill, and allowing for increased amounts and duration of drug application.

### 4.2 Future directions

Current, ongoing studies in our lab indicate that the addition of an electrode to the implanted pump allows for EEG measurement from healthy brain tissue surrounding the original tumor. As glioma cells do not generate electrical activity, impedance of the EEG signal by glioma tissue could be used to detect tumor recurrence. Our preliminary data already indicate that EEG signal strength is inversely proportional to the amount of surrounding tumor tissue. Repeated non-invasive MR brain imaging over the treatment course, along with paired EEG recordings, may allow for correlation of tumor size with EEG signal strength, and thus calibration of the EEG signal. This raises the interesting possibility that tumor recurrence could be monitored by the patient or physician via an app, replacing the need for MR imaging altogether. Chronically implanted (Ommaya) pumps are already used in glioblastoma patients, and chronically implanted electrodes have been in use for the treatment of movement disorders for many years — including newer adaptive algorithms: combining GBM treatment with diagnostic sensing is a logical extension of this.

While we tested two clinically used chemotherapeutic drugs separately, only BCNU is currently applied locally, in the form of a biodegradable polymer (Gliadel^®^) wafer. An emerging field in GBM treatment is the combined use of chemotherapy and immunotherapy, with reported synergistic effects; use of immunotherapeutic wafers is now being studied as a potential adjunct to chemotherapy (44). While we only tested one drug per pump, our dual-chamber pump allows for simultaneous application of two drugs, and future studies will address the efficacy of this approach.

The ultimate aim of this and future studies is translation to human GBM patients. This pump prototype would require surgical removal after a period of time. In order to facilitate the goal of permanent implantation, we will develop a biodegradable pump, using materials such as polycaprolactone, which can maintain structural integrity over the therapeutic lifetime of the device, and can be machine printed/molded according to the need of the specific patient. Future studies will evaluate a variety of biodegradable materials, with varying degradation characteristics, both *in vitro* and *in vivo*. Use of a *biodegradable pump* to apply BCNU might be expected to have a similar clinical efficacy and safety profile to biodegradable wafers. As this study indicates efficacy of local drug delivery using the current prototype, development of similar but biodegradable pumps will both enable long-term local single or dual drug treatment and timed pump dissolution, eliminating the need for surgical removal.

Development of permanently implanted, biodegradable devices, for local drug delivery, will require the integration of a number of disciplines, such as engineering, neuro-oncology, neurosurgery and neuroscience, and we and our engineering partners at the University of Utah are working on prototypes of an osmotic minipump which will biodegrade over a period of months to years, depending on the nature of the polymer. As treatments for GBM that prolong patient survival in a meaningful way are still lacking, the future of GBM treatment may lie in such cross-disciplinary collaborations, to develop novel treatments for this devastating disease.

## Data Availability

The original contributions presented in the study are included in the article/supplementary material. Further inquiries can be directed to the corresponding authors.
