# Correction: Efficacy of local convection enhanced delivery of chemotherapy using an intracerebral osmotic pump in a rat model of glioblastoma

**DOI:** 10.3389/fonc.2026.1828465

**Published:** 2026-03-24

**Authors:** John McDaid, Julian E. Bailes, Neilank K. Jha, George Bobustuc, Reed Berlet, Cassidy Kessinger, Eliana Whitcomb, John M. Lee, Daniil P. Aksenov, Matthew Walker, Azur Azapagic, Jade Bookwalter, Ata Ullah, Himanshu Sant, Jill Shea, Bruce K. Gale

**Affiliations:** 1 Department of Neurosurgery, Endeavor Health, Evanston, IL, United States; 2 Pritzker School of Medicine, Chicago, IL, United States; 3 Pritzker School of Medicine, University of Chicago, Chicago, IL, United States; 4 Deep Brain BCI Corp, Wilmington, DE, United States; 5 Intent Medical Group, Endeavor Health Advanced Neurosciences Institute, Arlington Heights, IL, United States; 6 Department of Pathology, Endeavor Health, Evanston, IL, United States; 7 Department of Radiology, Endeavor Health, Evanston, IL, United States; 8 Department of Anesthesiology, Endeavor Health, Evanston, IL, United States; 9 Department of Mechanical Engineering, The University of Utah, Salt Lake, UT, United States; 10 Department of Biomedical Engineering, The University of Utah, Salt Lake, UT, United States; 11 Department of Chemical Engineering, The University of Utah, Salt Lake, UT, United States; 12 Department of Surgery, University of Utah School of Medicine, Salt Lake, UT, United States

**Keywords:** brain, carmustine, direct delivery, osmotic pump, resection, temozolomide, tumor

An incorrect **Funding** statement was provided as funding for this project was not provided by NeuraSeed BCI but Deep Brain BCI.

The correct **Funding** statement is “The author(s) declared that financial support was received for this work and/or its publication. Funding for this project was provided by Deep Brain BCI. Advanced MRI capability was supported by NIH S10 OD034205 to DA.”.

The **Conflict of interest** statement was erroneously given as “NJ is Executive Chairman and CEO of NeuraSeed BCI. JEB is a noncompensated stockholder in Neuraseed BCI.”

The correct **Conflict of interest** statement is “NJ is Executive Chairman and CEO of Deep Brain BCI. JEB is a noncompensated stockholder in Deep Brain BCI.

The remaining author(s) declared that this work was conducted in the absence of any commercial or financial relationships that could be construed as a potential conflict of interest.

The authors declare that this study received funding from Deep Brain BCI. The funder was not involved in the study design; collection, analysis, interpretation of data; the writing of this article; or the decision to submit it for publication”.

The original version of this article has been updated.

